# Supplementary material for: Predicting visual function by interpreting a neuronal wiring diagram
Source: Nature. 2024 Oct 2;634(8032):113–23. doi: 10.1038/s41586-024-07953-5 (PMC11446822; doi:10.1038/s41586-024-07953-5)
Supplement: Supplementary file 6 — CRF and ERF predictions for individual TmY4 and TmY9 cells. Analogous to Supplementary Data 3, but for TmY target types. Shown are the top four monosynaptic pathways, the strongest pathway passing through each of the top ten intermediary types (ranking from Extended Data Fig. 7), and the trisynaptic pathway Tm1–TmY–Dm3–TmY (see the section entitled Prediction of spatial normalization). [file 41586_2024_7953_MOESM6_ESM.zip › DataS4/TmY9qΓèÑ/720575940641522421.pdf]

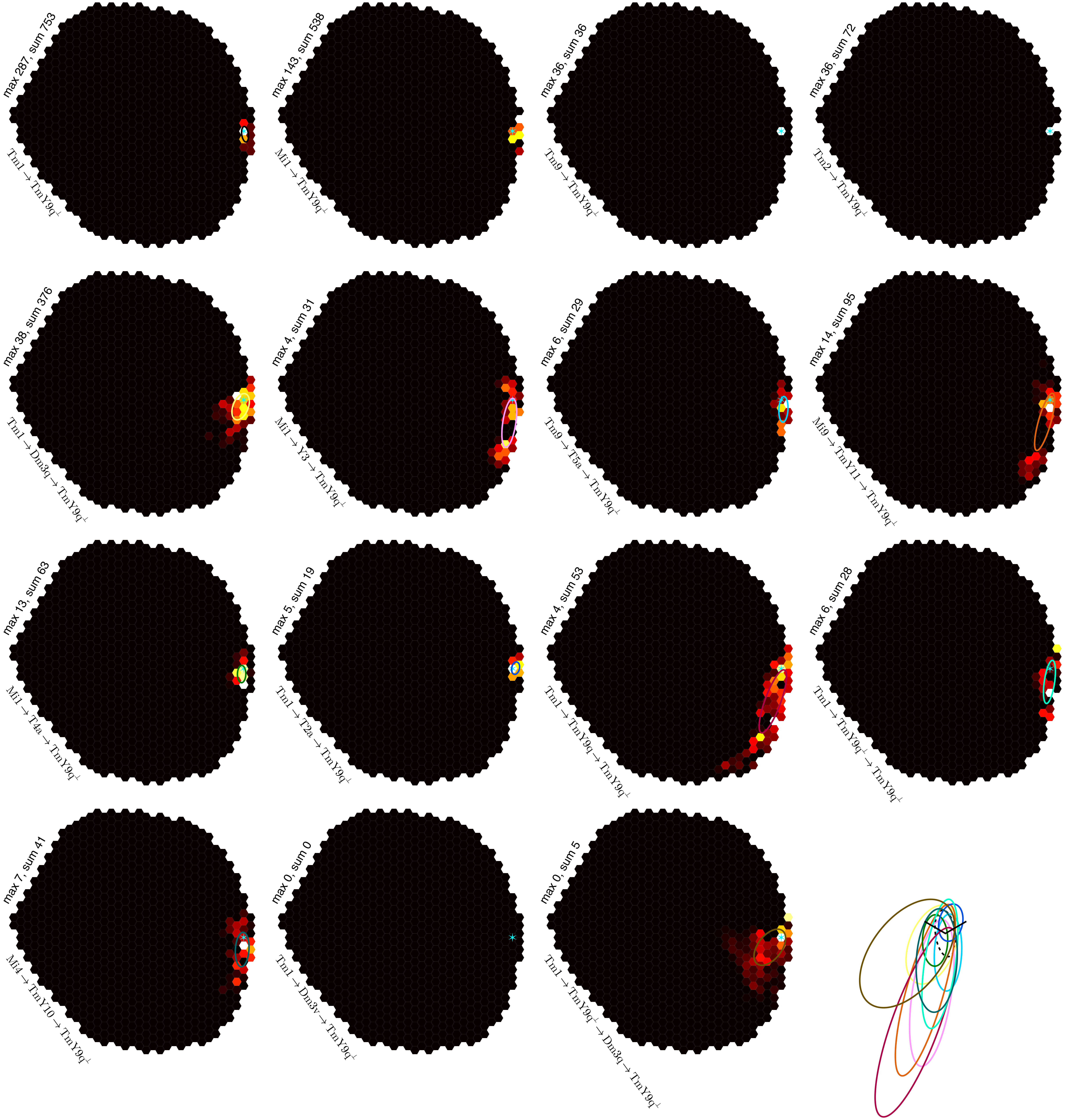

max 287, sum 753
Tm1 → TmY9q⊥
max 143, sum 538
Mi1 → TmY9q⊥
max 36, sum 36
Tm9 → TmY9q⊥
max 36, sum 72
Tm2 → TmY9q⊥
max 38, sum 376
Tm1 → Dm3q → TmY9q⊥
max 4, sum 31
Mi1 → Y3 → TmY9q⊥
max 6, sum 29
Tm9 → T5a → TmY9q⊥
max 14, sum 95
Mi9 → TmY11 → TmY9q⊥
max 13, sum 63
Mi1 → T4a → TmY9q⊥
max 5, sum 19
Tm1 → T2a → TmY9q⊥
max 4, sum 53
Tm1 → TmY9q → TmY9q⊥
max 6, sum 28
Tm1 → TmY9q⊥ → TmY9q⊥
max 7, sum 41
Mi4 → TmY10 → TmY9q⊥
max 0, sum 0
Tm1 → Dm3v → TmY9q⊥
max 0, sum 5
Tm1 → TmY9q⊥ → Dm3q → TmY9q⊥
